# Quantification of Citrullinated Histone H3 Bound DNA for Detection of Neutrophil Extracellular Traps

**DOI:** 10.3390/cancers12113424

**Published:** 2020-11-18

**Authors:** Marina Li, Cindy Lin, Aubrey Leso, Yulia Nefedova

**Affiliations:** Immunology, Microenvironment, and Metastasis Program, The Wistar Institute, Philadelphia, PA 19104, USA; marina.li@merck.com (M.L.); CLin@wistar.org (C.L.); aubrey.leso@vca.com (A.L.)

**Keywords:** neutrophil extracellular traps, NETs, NET quantitation, multiple myeloma, citrullinated histone H3

## Abstract

**Simple Summary:**

Neutrophil extracellular traps (NETs) are extracellular web-like structures comprised of proteins and DNA that have been associated with the development of cancer. Citrullination of histone H3 has been implicated in the formation of NETs. The aim of our study was to develop a quick and reliable method for NET quantitation. Here, we describe a novel protocol for the quantification of NETs based on the detection of citrullinated histone H3 bound to DNA (CitH3DNA binding assay). This assay was validated by comparing the ability of neutrophils from control tumor-free and myeloma-bearing mice to form NETs in response to stimuli. We demonstrated that neutrophils from tumor-bearing mice produced more NETs than those from tumor-free counterparts following stimulation with PMA. The increase in NET production as detected by microscopy correlated with significantly higher histone H3 citrullination levels and increased measurements of CitH3DNA in our novel binding assay.

**Abstract:**

Formation of neutrophil extracellular traps (NETs) has been associated with multiple pathologies including cancer. While the visualization of NETs by microscopy is a routine technique, their quantification presents a number of challenges. Commonly, as citrullination of histone H3 is required for NET formation, the presence of this modified histone along with DNA is considered to be a hallmark of NETs. Here, we describe and validate a novel assay for the quantification of NETs based on the detection of citrullinated histone H3 bound to DNA (CitH3DNA binding assay). Using this assay, we investigated the effect of phorbol 12-myristate 13-acetate (PMA) on NET formation by neutrophils isolated from the bone marrow of control and myeloma-bearing mice. We found that PMA induced citrullination of histone H3, an increase in the level of CitH3DNA, and NET formation in neutrophils from both tumor-free and myeloma-bearing mice. The levels of CitH3DNA in the NET fractions, as measured by our assay directly correlated with the citrullination of histone H3 in neutrophils, as detected by Western blotting, and were significantly higher in PMA-stimulated compared to unstimulated neutrophils. Neutrophils from tumor-bearing mice produced more NETs than those from tumor-free counterparts following stimulation with PMA. The increase in NET production correlated with significantly higher histone H3 citrullination levels and increased measurements of CitH3DNA. Thus, our data indicate that bone marrow neutrophils from myeloma-bearing hosts are prone to NET formation.

## 1. Introduction

There has been recent increased interest in the evaluation of neutrophil extracellular traps (NETs) as there is a growing body of evidence that they are involved in a number of pathologies including cancer [1,2,3,4]. Formation of NETs by neutrophils was first described by Brinkmann, Zychlinsky, and colleagues in 2004 [5]. NETs are extracellular, web-like structures comprised of fibers of chromatin decorated with proteins and peptides most of which are normally found in the granules and cytoplasm of neutrophils [5,6]. NETs are produced in a process called NETosis in response to the variety of stimuli including calcium ionophore, phorbol 12-myristate 13-acetate (PMA), lipopolysaccharide (LPS), inflammatory cytokines such as interleukin (IL)-8, granulocyte colony stimulating factor, activated platelets, and others [5,6,7,8,9,10]. A number of molecular events leading to NETosis has been identified (reviewed in [11]). PMA is a chemical tool that is commonly used as a potent NET inducer in mechanistic studies of NET formation. PMA-induced NETosis is initiated by the activation of protein kinase C, the release of calcium from intracellular stores, and activation of the Mitogen-activated protein kinase-Extracellular-signal-regulated kinase (Raf–MEK–ERK) pathway followed by assembly of nicotinamide adenine dinucleotide phosphate (NADPH) oxidase and generation of reactive oxygen species including hydrogen peroxide [6]. Hydrogen peroxide triggers the activation of neutrophil elastase (NE) and myeloperoxidase (MPO) followed by their subsequent mobilization from azurophilic granules and translocation to the nucleus where they digest core histones promoting chromatin decondensation [12,13]. Simultaneously, increased intracellular calcium levels lead to the activation of peptidyl arginine deiminase 4 (PAD4), an enzyme responsible for citrullination. Citrullination is a posttranslational modification that converts arginine residues into citrulline. PAD4 then translocates from the cytoplasm to the nucleus and mediates citrullination of histones. Citrullination of histone H3 at arginine residues R2, R8, and R17 by PAD4 results in a reduction of the positive charge and decondensation of chromatin [14,15,16]. The nuclei of neutrophils then lose their characteristic lobular structure, round up and expand. This is followed by disintegration of the nuclear membrane resulting in mixing of the nucleoplasm and cytoplasm. Finally, the cell membrane ruptures, and the contents of the neutrophil is released into the extracellular space in the form of a NET. 

Citrullinated histone H3 bound to cell-free DNA is considered a hallmark of NETs. The presence of citrullinated histone H3 (H3cit) has been documented in NETs derived from neutrophils from tumor-bearing hosts, hosts with bacterial infections, and in vitro in response to varying stimuli [15,17]. Neutrophils from PAD4 knockout mice have defects in producing NETs [14]. 

Increased NET formation has been associated with the growth of solid tumors [1,3,18,19,20]. Multiple myeloma is a cancer of plasma cells that develops and preferentially grows in the bone marrow (BM). Recently, we have demonstrated that pharmacological blockade of NET formation prolonged the survival of myeloma-bearing mice [21]. However, whether myeloma tumors pre-condition neutrophils for NET formation has not been investigated yet. 

Several techniques have been used for NET detection including immunofluorescence microscopy, enzyme-linked immunosorbent assays (ELISA), and flow cytometry [5,6,22,23,24,25,26,27,28,29,30]. Although these techniques improved our understanding of the role played by NETs in different pathologies, the lack of standardization of the assays rendered any quantitative comparison of the results difficult. 

Here, we describe and validate a novel assay for the detection of DNA specifically bound to the citrullinated histone H3 in NETs. This assay allows quick detection and quantification of NETs. Using this assay, we evaluated the ability of neutrophils from BM of myeloma-bearing mice to form NETs. Our data demonstrated that neutrophils from myeloma-bearing mice were conditioned to form significantly more NETs in response to stimulation with PMA than neutrophils from tumor-free mice. 

## 2. Results

### 2.1. PMA Induces NET Formation

**Figure 1:**
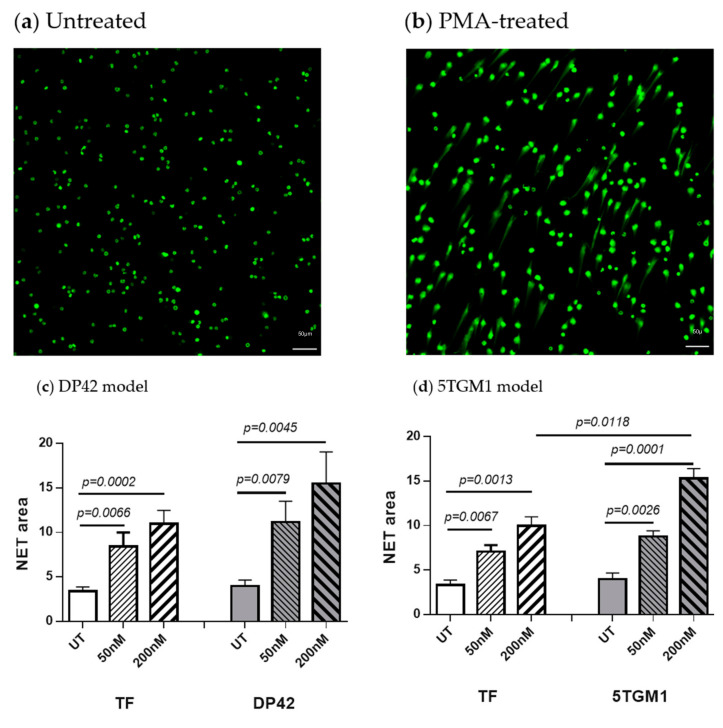
Neutrophil extracellular trap (NET) formation induced by phorbol 12-myristate 13-acetate (PMA). Neutrophils were isolated from the BM of DP42-bearing mice, 5TGM1-bearing mice, or control tumor-free (TF) mice and stimulated in vitro with 50 nM or 200 nM PMA for 4 h. Formation of NETs was evaluated by microscopy and quantified. (**a**,**b**) Representative images of untreated (**a**) or stimulated with PMA (**b**) neutrophils. (**c**,**d**) Quantitation of NET area for neutrophils from DP42-bearing (*n* = 12) and tumor-free (*n* = 9) mice (**c**) and 5TGM1-bearing (*n* = 4) and tumor-free (*n* = 4) mice (**d**). Mean ± SEM values are shown. UT—untreated.

We compared the ability of neutrophils isolated from the BM of tumor-free and myeloma-bearing mice to form NETs in response to stimulation with PMA. Myeloma tumors were established in syngeneic mice by intravenous injection of DP42 or 5TGM1 cells. The presence of NETs was evaluated by microscopy following staining with SYTOX Green Nucleic Acid Stain (ThermoFisher Scientific, Waltham, MA, USA). BM neutrophils from both control and myeloma-bearing mice were able to spontaneously form NETs; however, the level of spontaneous NETosis was similarly low between neutrophils from myeloma-bearing and tumor-free mice (Figure 1). As expected, PMA dramatically and in a dose-dependent manner induced NET formation. Interestingly, in response to PMA, neutrophils from both DP42- and 5TGM1-bearing mice upregulated their NET production substantially more than those from control mice (Figure 1c,d, respectively). 

### 2.2. PMA Induces Citrullination of Histone H3 in Neutrophils

**Figure 2:**
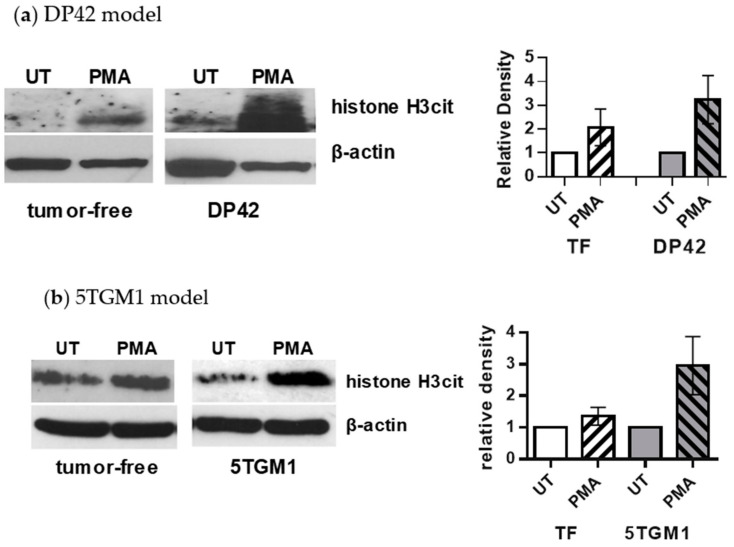
Level of citrullinated histone H3 in PMA-stimulated neutrophils from control and myeloma-bearing mice. Neutrophils were isolated from the BM of (**a**) DP42 myeloma-bearing or control tumor-free mice (*n* = 5 and *n* = 4, respectively) or (**b**) 5TGM1-bearing or control tumor-free mice (*n* = 3 for each group) and stimulated with 200 nM PMA for 4 h. Cells were then collected and subjected to Western blotting with antibodies against citrullinated histone H3. Equal loading was confirmed by re-probing membranes with antibodies against β-actin. Western blots are shown in Figure S1. Left, representative Western blots are shown. Right, densitometry results presented as mean ± SEM values are shown.

Next, we investigated whether citrullination of histone H3 is associated with PMA-induced NET formation. Neutrophils were isolated from the BM of control tumor-free mice or DP42- or 5TGM1-bearing mice and stimulated in vitro with 200 nM PMA followed by detection of citrullinated histone H3 using Western blotting. A low expression of histone H3cit was detected in unstimulated neutrophils from both tumor-free and myeloma-bearing mice. PMA-induced citrullination of histone H3 was significantly higher in neutrophils from DP42 (Figure 2a) or 5TGM1 (Figure 2b) myeloma-bearing mice compared to control tumor-free mice. Thus, these data demonstrate that the level of citrullinated histone H3 directly correlates with the amount of NETs produced by neutrophils upon stimulation with PMA. Quantification of NETs is a laborious process requiring advanced instrumentation. Therefore, we developed an assay allowing the quick and quantitative detection of NETs.

### 2.3. Development of a CitH3DNA Binding Assay for Detection of NETs

**Figure 3:**
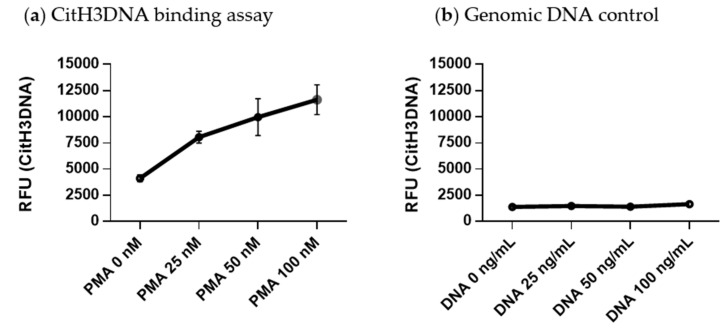
Quantitative citrullinated histone H3 bound to DNA (CitH3DNA) binding assay for detection of NETs. High binding 96-well plates were coated with anti-histone H3cit antibodies. (**a**) Neutrophils were isolated from BM of myeloma-bearing mice and stimulated in vitro with indicated concentrations of PMA for 4 h. NET fractions were collected from each condition and 100 μL were loaded per well. (**b**) Indicated amount of genomic DNA was added per well. (**a**,**b**) Fluorescence of SYTOX Green nucleic acid stain was measured using a Victor X3 plate reader. RFU—relative fluorescence units.

Neutrophils were isolated from the BM of myeloma-bearing mice and stimulated with 25–100 nM PMA for 4 h. Culture media was then removed, and neutrophils were incubated in harvesting buffer followed by centrifugation. Pelleted cells were removed whilst supernatant that represented the NET fraction was collected and used for subsequent analysis. Black high binding 96-well plates (Cat. #655077, Greiner bio-one, Monroe, NC, USA) were coated with 1.5 μg/mL of antibodies against citrullinated histone H3 (citrulline R2+R8+R17; Cat. #5103, Abcam, Cambridge, MA, USA) diluted in phosphate-buffered saline (PBS) (200 μL/well) and incubated overnight at 4 °C. After that time, plates were washed three times with 0.05% Tween-20 in PBS (PBST) prior to blocking with 5% bovine serum albumin in PBS (200 μL/well) for 2 h at room temperature. The plates were then washed three times with PBST, and 100 μL of the NET fraction collected from each condition was added per well. To ensure the specificity of chromatin detection and exclude non-specific binding of cell-free DNA, 25–100 ng/mL of genomic DNA was added per well as a negative control. The plates were incubated for 4 h at room temperature while shaking and then washed three times with PBST prior to adding SYTOX Green nucleic acid stain (Cat. #S7020, ThermoFisher Scientific, Waltham, MA, USA) diluted in HBSS to a final concentration of 1 μM (100 μL/well). The fluorescence of SYTOX Green was measured using a Victor X3 plate reader (PerkinElmer, Waltham, MA, USA). We refer to this assay as the citrullinated histone H3 bound to DNA (CitH3DNA) binding assay. The stimulation of neutrophils with PMA resulted in a dose-dependent increase in the level of cell-free DNA bound to citrullinated histone H3 in the NET fraction (Figure 3a). Genomic DNA added at increasing concentrations did not bind to citrullinated histone H3 (Figure 3b), confirming the specificity of the assay for detecting only modified chromatin.

### 2.4. Detection of Neutrophil-Derived Citrullinated Histone H3 Bound to Cell-Free DNA 

**Figure 4:**
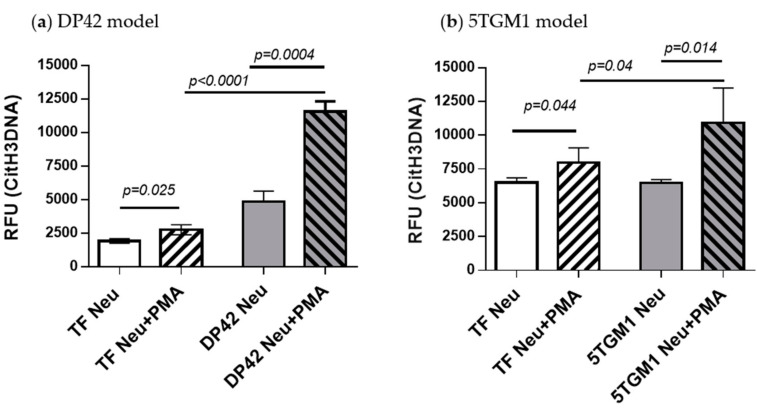
Detection of neutrophil-derived citrullinated histone H3-bound DNA produced in response to stimulation with PMA. Neutrophils were isolated from the BM of (**a**) DP42-bearing mice, (**b**) 5TGM1-bearing mice, or control tumor-free mice and stimulated with 200 nM PMA for 4 h. The NET fractions were then isolated and used in the CitH3DNA binding assay. Combined data from 3 independent experiments are shown. Mean ± SD values are shown. RFU—relative fluorescence units.

To validate the CitH3DNA binding assay, we used this assay for the detection of PMA-stimulated NETs produced by BM neutrophils from tumor-free or myeloma-bearing mice. As anticipated, an increased level of cell-free DNA bound to citrullinated histone H3 was found in NET fractions from neutrophils from both tumor-free and DP42 myeloma-bearing mice in response to stimulation with PMA (Figure 4a). A similar effect was observed in neutrophils isolated from 5TGM1-bearing mice or their control counterparts (Figure 4b). This effect was significantly more pronounced in neutrophils from myeloma-bearing mice compared to neutrophils from tumor-free mice (Figure 4). The results of the CitH3DNA assay are consistent with our Western blotting data, showing a dramatic increase in citrullinated histone H3 level in PMA-stimulated neutrophils from DP42- and 5TGM1-bearing mice compared to control mice (Figure 2). 

Taken together, our data demonstrate that, in the myeloma BM microenvironment, neutrophils are more prone to NET formation in response to stimulation with PMA compared to neutrophils from tumor-free mice. 

### 2.5. Detection of IL-1β-Induced NETosis

**Figure 5:**
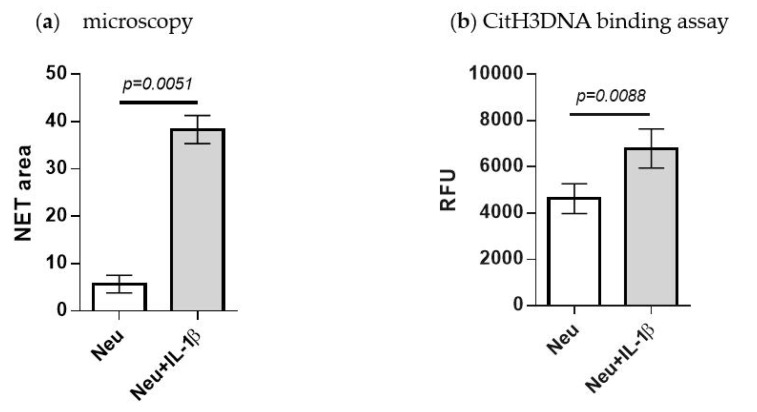
Quantification of citrullinated histone H3 for detection of IL-1β-induced NETosis. Neutrophils were isolated from the BM of tumor-free mice (*n* = 3) and stimulated in vitro with 200 ng/mL of murine IL-1β for 6 h. (**a**) Formation of NETs was evaluated by microscopy and quantified. (**b**) NET fractions were collected and used in the CitH3DNA binding assay. Mean ± SEM values obtained from neutrophils isolated from 3 individual mice are shown.

A number of cytokines are elevated in the myeloma bone marrow microenvironment and some of them, including IL-1β, have been also reported to induce NETosis. We evaluated whether the CitH3DNA binding assay could represent a valuable tool for detection of NETosis induced by physiological stimuli and utilized IL-1β for these studies. Neutrophils were isolated from the bone marrow and treated with mouse recombinant IL-1β for 6 h followed by microscopy analysis of NET formation. As shown in Figure 5a, addition of IL-1β stimulated neutrophils to produce NETs. In parallel, we performed a CitH3DNA binding assay. Treatment of neutrophils with IL-1β led to a significantly increased level of citrullinated histone H3 bound to extracellular DNA (Figure 5b). These data demonstrate that the CitH3DNA binding assay represents a useful method for the evaluation of NETosis. 

## 3. Discussion

Since the discovery of NETs, the understanding of their role in pathological conditions has become the subject of a growing number of studies [5]. This has generated a need for developing techniques of NET assessment. Here, we describe the development of a specific, quick, objective, and cost-effective fluorescent assay – the CitH3DNA binding assay – to quantify NETs. This assay utilizes an antibody specific for citrullinated histone H3, one of the NET markers, as the capture antibody, followed by a fluorescent nucleic acid dye SYTOX Green, which is impermeable to live cells and thus labels only cell-free DNA, Using our CitH3DNA assay, we demonstrated that the level of histone H3cit bound to cell-free DNA in the NET fraction directly correlates with the level of intracellular citrullinated histone H3 in neutrophils and the amount of NETs produced by these neutrophils. 

Several techniques for the evaluation of NET formation have been previously developed and in use now; however, each of them has limitations. The presence of cell-free DNA was proposed to be associated with the presence of NETs and a common method used to detect cell-free DNA in the supernatant employs a cell-impermeable fluorescent dye such as SYTOX Green [31,32]. However, the major limitation for this method is the lack of specificity, as DNA released as a result of other forms of cell death (e.g., necrosis, apoptosis) can also be detected by the dye. Our binding assay allows detection of only histone H3cit-bound cell-free DNA and therefore, it is specific for evaluation of NETosis. Various sandwich ELISAs have also been developed to detect extracellular DNA complexed with NET-specific proteins such as MPO or NE [22,23,26,33,34]. However, it has been shown that the release of NE and MPO is not strictly specific to NETosis. NE can be secreted by neutrophils and macrophages during inflammation [35] and MPO can be released into the extracellular space during degranulation [36]. Both of these proteins are highly cationic [37,38] and can bind to cell-free DNA originating from apoptotic or necrotic cells, thus questioning their specificity as NET markers. In this respect, our assay seems to be superior, as citrullination of histone H3 has been reported to be associated specifically with NETosis. Recently, Thalin et al. described a new ELISA to quantify histone H3cit levels in human plasma [27]. While this assay can detect histone H3cit, it is not specific for histone H3cit bound to DNA. Since histones can be released into the extracellular space not only by neutrophils as a part of NETs but also by damaged or activated cells [39] this assay can only be used for NET detection in combination with other methods. Our CitH3DNA assay is specific for detection of histone H3cit bound to cell-free DNA and therefore, superior in its specificity of detection NETs. Other methods of NET evaluation that are based on immunofluorescence or confocal microscopy detect extracellular DNA co-localized with neutrophil-derived proteins (e.g., MPO, NE, H3cit) [6,29,40]. While these methods are more specific, they are laborious and susceptible to observer bias. In recent years, automated NET-based fluorescence microscopy assays have minimized observer subjectivity and facilitated the acquisition and quantification of NETs [41,42]. While these assays are indeed more robust, they require considerable expertise and advanced instrumentation. Flow cytometry has also been proposed as a method to monitor cell-appendant NET components [24,25] and a swelling of the nucleus in NETting neutrophils [28]. However, the latter approach appears to be complex and requires specific instrumentation (e.g., ImageStream Multispectral Imaging Flow Cytometer). Furthermore, the reliability of flow cytometry for the evaluation of NET-forming neutrophils is debatable as one of the major concerns is that flow cytometry requires cells to be in suspension even though cells undergoing NETosis are bound to a substrate and form clumps due to the high viscosity of DNA. The intracellular level of citrullinated histone H3 in circulating neutrophils could be detected by flow cytometry [43]; however, it does not represent a direct measurement of NETs. Our binding assay is objective, quantitative, robust, and does not require advanced instrumentation as compared to detection of NETs by microscopy or flow cytometry. 

Recently, an increasing number of studies have reported the presence of NETs and described their role in the progression of different types of solid tumors (reviewed in [44]). We recently demonstrated that myeloma cells induce NETosis and that pharmacologically targeting PAD4 has an anti-tumor effect in mouse model of myeloma [21]. However, the question of whether neutrophils in the myeloma BM microenvironment are primed to produce NETs remains unanswered. Here, we addressed this question by comparing the NET-forming ability of neutrophils from tumor-free and myeloma-bearing mice in response to PMA, a chemical agent frequently used as a potent NET inducer in mechanistic studies of NET formation [12]. PMA-stimulated neutrophils from tumor-free mice did produce NETs but demonstrated only a moderate increase in intracellular levels of histone H3cit and a very moderate but statistically significant increase in CitH3DNA in the NET fraction. Similar results were obtained for neutrophils stimulated with IL-1β, a cytokine present in the bone marrow microenvironment. These results are consistent with published data demonstrating that the PMA-induced increase in the level of histone H3cit was low in neutrophils isolated from peripheral blood of healthy donors [15,45,46]. PMA-stimulated neutrophils from myeloma-bearing mice produced more NETs compared to control neutrophils (Figure 1). Consistently, levels of both intracellular histone H3cit and CitH3DNA in the NET fraction from myeloma-bearing mice were dramatically increased upon PMA stimulation compared to controls. These data suggest that the myeloma BM microenvironment is altering neutrophil responses to NET-inducing stimuli. Future work will be needed to identify the underlying mechanism of this apparent pre-conditioning of neutrophils for NET formation by myeloma tumors. One potential explanation is the elevated level of reactive oxygen species reported to be produced by neutrophils from tumor-bearing hosts [47]. Alternatively, there is a possibility that neutrophils from myeloma-bearing mice have elevated levels and/or activity of PAD4, the enzyme responsible for citrullination of histone H3, compared to neutrophils from tumor-free mice. 

In conclusion, we have developed a specific, quick, objective, and cost-effective fluorescent binding assay to quantify NETs by measuring the levels of the NET biomarker histone H3cit bound to cell-free DNA. Using this CitH3DNA binding assay, we demonstrated that BM neutrophils produced NETs in response to stimulation with PMA and that neutrophils from myeloma-bearing mice are prone to NET production compared to neutrophils from tumor-free mice. 

## 4. Materials and Methods 

### 4.1. Cell Culture 

The mouse multiple myeloma cell lines DP42 and 5TGM1 were gifts from Dr. Van Ness (University of Minnesota, Minneapolis, MN, USA) and Dr. Babatunde Oyajobi (The University of Texas Health Science Center at San Antonio, TX, USA), respectively. Cells were maintained in RPMI-1640 medium supplemented with L-glutamine, 10% fetal bovine serum (FBS), and 1× Penicillin-Streptomycin. DP42 cells were also supplemented with 0.5 ng/mL murine IL-6 (Cat # 406-ML, R&D Systems, Minneapolis, MN, USA).

### 4.2. Mouse Model of Multiple Myeloma

All animal experiments were carried out in accordance with institutional guidelines and were approved by the Institutional Animal Care and Use Committee of The Wistar Institute (Animal Welfare Assurance ID A3432-01). C57BL/6 and FVB/N mice were purchased from Charles River Laboratories and were crossed to obtain F1 progeny of mixed background. In these mice, myeloma tumors were established by inoculation of 5 × 10^3^ DP42 cells intravenously (i.v.) into the tail vein. Mice were euthanized 12 days after tumor cell injection. KaLwRij mice were kindly provided by Dr. Lori Hazlehurst (West Virginia University, Morgantown, WV, USA). 5TGM1 tumors were established by inoculation of 1 × 10^6^ 5TGM1 cells i.v. into the tail vein; mice were euthanized 18 days after this. The femur and tibia were collected and used as a source of BM cells. In the control, the femur and tibia were collected from tumor-free mice. 

### 4.3. Isolation of Mouse Neutrophils

Neutrophils were isolated from the BM using magnetic-activated cell sorting (MACS) technology (Miltenyi Biotec, Auburn, CA, USA). Briefly, BM cells were labeled with the biotin-Ly6G antibody (Cat #130-101-884), then washed with MACS buffer (1% FBS and 5 mM ethylenediaminetetraacetic acid (EDTA) in PBS) and incubated with streptavidin conjugated microbeads (Cat #130-048-101) according to the manufacturer’s protocol. Labeled cells were eluted from the LS column (Cat #130-042-401) for positive selection. The purity of isolated Ly6G cells was 98% or more as determined by flow cytometry. All Ly6G^+^ cells also expressed the CD11b marker.

### 4.4. Induction and Isolation of NETs 

Mouse BM neutrophils were plated in a flat-bottom 6-well tissue culture plate (Cat #130184, ThermoFisher Scientific) at a density of 2 × 10^6^ cells/mL. The cells were stimulated with 25–200 nM PMA (Cat # P8139, Sigma-Aldrich, St. Louis, MO, USA) for 4 h. For conditions using cytokines, cells were stimulated with 200 ng/mL of IL-1β (Peprotech, Rocky Hill, NJ, USA Cat #211-11B) for 6 h. After that time, the medium in the well was pipetted out carefully to avoid disturbing the NETs formed. Each well was rinsed quickly with HBSS and 300 μL of harvesting buffer (10 mM MgCl2 in RPMI + 1 unit/mL DNase I (Cat #D5025, Sigma-Aldrich)) was added per well and incubated for 20 min at 37 °C followed by the addition of EDTA (to a final concentration of 5 mM, Cat #15575-038, Invitrogen, Carlsbad, CA, USA) to halt the DNase reaction. The content of the well was then collected by vigorous pipetting and spun for 5 min at 300× *g*. Collected supernatant represented the NET fraction. 

### 4.5. NET Detection by Microscopy 

Mouse BM neutrophils were placed in flat-bottom 24-well tissue culture plate and stimulated with 50 nM or 200 nM PMA for 4 h. Neutrophils were then fixed with 4% paraformaldehyde (Electron Microscopy Sciences, Hatfield, PA, USA) followed by staining with SYTOX Green Nucleic Acid Stain (SYTOX) at a final concentration of 250 nM. A Nikon Te300 inverted microscope equipped with a motorized XY stage was used to image NETs. Twenty-five images were acquired per well selected from random locations. The z-stacks per location were then combined into an (EDF) extended depth focused image. The total NET area was calculated by segmenting each image using a defined threshold pixel intensity setting. The spot detection tool in NIS-Elements Ar was used to count the number of cells per field. The sum of the total NET area in the 25 random fields of view was divided by the total number of cells in the 25 fields of view to obtain the NET area (µM)/cell. 

### 4.6. Western Blotting

Neutrophils were lysed using radioimmunoprecipitation assay buffer supplemented with 1 mM EDTA and 1× Halt protease and phosphatase inhibitor (ThermoFisher Scientific). Membranes were blocked with 5% nonfat dry milk and incubated with anti-histone H3 (citrulline R2+R8+R17) (Cat. #5103, Abcam, dilution 1:1000) antibody followed by incubation with secondary horseradish peroxidase-conjugated antibodies. Membranes were probed with antibodies against β-actin (Cat. #sc-47778, Santa Cruz Biotechnology, Dallas, TX, USA dilution 1:5000) to ensure equal loading. Densitometry of specific bands was performed using ImageJ software. The intensity of the citrullinated histone H3 band was normalized to the intensity of corresponding β-actin band. Data were presented as the fold change in PMA-stimulated neutrophils compared to untreated neutrophils. 

### 4.7. Statistical Analysis 

Statistical analysis was performed using GraphPad Prism 5 software (GraphPad Software Inc, San Diego, CA, USA). The two-tailed Student *t* test was used to determine the differences between groups. A *p*-value of less than 0.05 was considered statistically significant. 

## 5. Conclusions

In conclusion, we developed and validated a quick, objective, and cost-effective fluorescent binding assay to quantify NETs by measuring the level of the NET biomarker histone H3cit bound to cell-free DNA. Using this CitH3DNA binding assay, we demonstrated that neutrophils from both tumor-free and myeloma-bearing mice form NETs in response to stimulation by PMA. However, neutrophils from myeloma-bearing mice are prone to higher levels of NET production compared to neutrophils from tumor-free mice. 

## Supplementary Materials

The following are available online at https://www.mdpi.com/2072-6694/12/11/3424/s1, Figure S1: Evaluation of histone H3cit in PMA-stimulated neutrophils.
