# Supplementary material for: Quantification of Citrullinated Histone H3 Bound DNA for Detection of Neutrophil Extracellular Traps
Source: Cancers (Basel). 2020 Nov 18;12(11):3424. doi: 10.3390/cancers12113424 (PMC7698949; doi:10.3390/cancers12113424)
Supplement: Supplementary file 1 [file cancers-12-03424-s001.pdf]

Supplementary Materials:

Quantification of Citrullinated Histone H3 Bound DNA for Detection of Neutrophil Extracellular Traps

Marina Li, Cindy Lin, Aubrey Leso and Yulia Nefedova

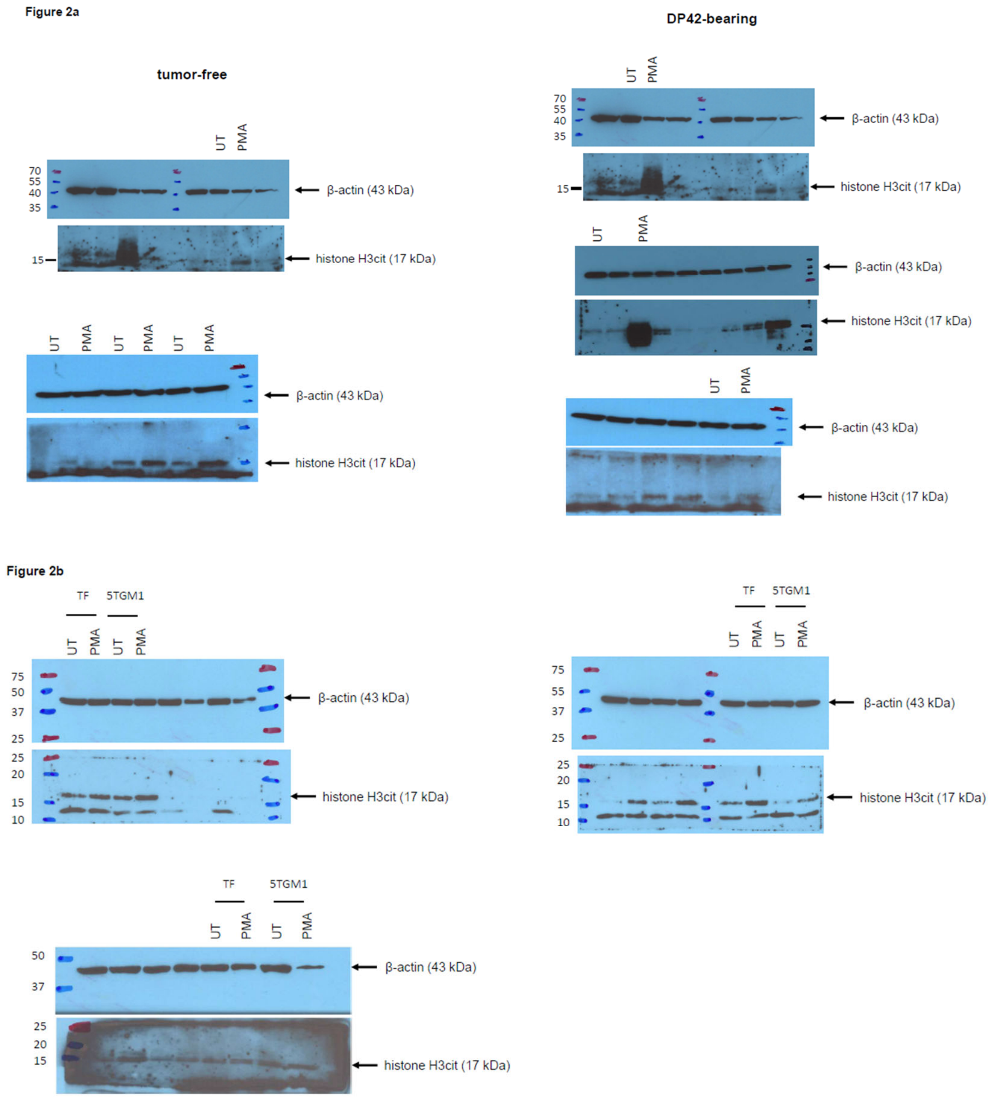

Figure S1. Evaluation of histone H3cit in PMA-stimulated neutrophils. Neutrophils were isolated from the BM of DP42 myeloma-bearing or control tumor-free mice or 5TGM1-bearing or tumor-free (TF) mice and left untreated (UT) or stimulated with 200 nM PMA for 4 h. Cells were then collected and subjected to Western blotting with antibodies against citrullinated histone H3. Equal loading was confirmed by re-probing membranes with antibodies against β-actin.
